# Clear cell renal cell carcinoma with cystic component similar to multilocular cystic renal neoplasm of low malignant potential: a rare pattern of cyst-dependent progression from multilocular cystic renal neoplasm of low malignant potential

**DOI:** 10.1186/s13000-023-01315-x

**Published:** 2023-02-21

**Authors:** Bo Yang, Lin Sun, Wen-feng Cao, Li-sha Qi, Yu-hong Guo, Yan Sun

**Affiliations:** Department of Pathology, Tianjin Medical University Cancer Institute and Hospital, National Clinical Research Center for Cancer, Key Laboratory of Cancer Prevention and Therapy, Tianjin, Tianjin’s Clinical Research Center for Cancer, Tianjin, China, Huanhu West Road, Hexi District, Tianjin, 300060 China

**Keywords:** Multilocular cystic renal neoplasm of low malignant potential, Cystic component, Renal cell carcinoma, Immunohistochemistry, Prognosis

## Abstract

**Background:**

For clear cell renal cell carcinoma (ccRCC) with cystic component similar to multilocular cystic renal neoplasm of low malignant potential (MCRN-LMP) and solid low-grade component simultaneously, we propose the designation “ccRCC with cystic component similar to MCRN-LMP” and to study the relationship between MCRN-LMP and it.

**Methods:**

Twelve cases of MCRN-LMP and 33 cases of ccRCC with cystic component similar to MCRN-LMP were collected from 3,265 consecutive RCCs to compare them in clinicopathological features, immunohistochemical findings (PAX8, CA-IX, CK7, Vimentin, CD10, P504s, TFE3, 34βE12) and prognosis.

**Results:**

There was no significant difference in age, sex ratio, tumor size, treatment, grade and stage between them (*P* > 0.05). All ccRCCs with cystic component similar to MCRN-LMP coexisted with MCRN-LMP and solid low-grade ccRCCs, and MCRN-LMP component ranged from 20 to 90% (median, 59%). The positive ratio of CK7 and 34βE12 in MCRN-LMPs and ccRCCs’ cystic parts was significantly higher than that in ccRCCs’ solid parts, but the positive ratio of CD10 in MCRN-LMPs and ccRCCs’ cystic parts was significantly lower than that in ccRCCs’ solid parts (*P* < 0.05). There was no significant difference of all immunohistochemistry profiles between MCRN-LMPs and ccRCCs’ cystic parts (*P* > 0.05). No patient developed recurrence or metastasis.

**Conclusions:**

MCRN-LMP and ccRCC with cystic component similar to MCRN-LMP have similarity and homology in clinicopathological features, immunohistochemical findings and prognosis, and form a low-grade spectrum with indolent or low malignant potential behavior. The ccRCC with cystic component similar to MCRN-LMP may be a rare pattern of cyst-dependent progression from MCRN-LMP.

## Background

Multilocular cystic renal neoplasm of low malignant potential (MCRN-LMP) is a tumor composed entirely of numerous cysts, the septa of which contain individual or groups of clear cells without expansile growth, and that is morphologically indistinguishable from low-grade clear cell renal cell carcinoma (ccRCC), but recurrence or metastasis have not been reported [1, 2]. The neoplastic cells are strongly immunoreactive to PAX8 and carbonic anhydrase IX (CA-IX), and more frequently expressed CK7 [1, 3]. Some Xp11 translocation RCCs with *MED15*-*TFE3* fusion have been described containing extensive cystic architecture similar to MCRN-LMP with TFE3 positive immunostaining [4–6]. Therefore, the diagnosis of MCRN-LMP needs to strictly follow the criteria of morphology and immunohistochemistry (IHC) staining.

We have encountered some low-grade ccRCCs (WHO/International Society of Urological Pathology (ISUP) grade 1 or 2) with cystic component similar to MCRN-LMP and solid epithelial component simultaneously, as mentioned in some studies [7–9]. We designate these tumors “ccRCC with cystic component similar to MCRN-LMP”. Genetic studies have clearly linked ccRCC and MCRN-LMP, with 74% chromosome 3p deletions and 25% von Hippel–Lindau (*VHL*) mutations in MCRN-LMP cases, similar to ccRCC [10, 11]. However, there is no relevant study on whether ccRCC with cystic component similar to MCRN-LMP originates from MCRN-LMP. In order to shed light on the relationship between MCRN-LMP and ccRCC with cystic component similar to MCRN-LMP, we collected 12 cases of MCRN-LMP and 33 cases of ccRCC with cystic component similar to MCRN-LMP from 3,265 consecutive RCCs to analyze their similarities and differences in clinicopathological features, immunohistochemical findings and prognosis.

## Materials and methods

### Case selection

We designated “ccRCC with cystic component similar to MCRN-LMP” according to the following criteria: 1) the solid component was low-grade ccRCC (WHO/ ISUP grade 1 or 2); 2) the range of cystic component similar to MCRN-LMP was at least 20%; 3) excluding extensive coagulative (cystic) necrosis; 4) the minimum diameter of individual cysts in cystic component similar to MCRN-LMP was 1 mm; 5) the minimum diameter of cystic component similar to MCRN-LMP was 5 mm. We retrospectively collected the 3,265 consecutive RCCs which underwent partial or radical nephrectomy and had the IHC staining of a panel (PAX8, CD10, CA-IX, Vimentin, CK7, CD117, P504s, TFE3) at Tianjin Medical University Cancer Institute & Hospital from January 2012 to December 2020. The hematoxylin & eosin (H&E) and IHC staining slides were reviewed independently by experienced pathologists (Y.B, Q.L.S and C.W.F). Finally, 2,901 (88.9%) cases were diagnosed as clear cell RCC, and 12 (0.4%) cases were diagnosed as MCRN-LMP. In 2,901 cases of clear cell RCCs, 33 (1.1%) cases were ccRCCs with cystic component similar to MCRN-LMP. The clinicopathological features of all cases were collected, and all patients were followed until January 2022. This study was approved by the Ethical Review Committee of Tianjin Medical University Cancer Institute & Hospital (Approval No: bc2022136).

### IHC

Tumor tissues were fixed in 10% formalin and embedded in paraffin. The 4-μm-thick whole sections were performed IHC staining with an automated Ventana BenchMark XT system (Roche, Ventana Medical Systems Inc., Tucson) for the following antibodies: PAX8 (4H7B3, 1:100; ProteinTech Group, Rosemont, IL), CA-IX (ab1508, 1:1000; Abcam), CK7 (OV-TL12/30, prediluted; MXB Biotechnologies), Vimentin (V9, prediluted; MXB Biotechnologies), CD10 (56C6, prediluted; MXB Biotechnologies), P504s (13H4, prediluted; MXB Biotechnologies), TFE3 (SC-5958, 1:300; Santa Cruz, CA), 34βE12 (prediluted; MXB Biotechnologies). Positive and negative controls yielded appropriate results for each antibody.

Immunoreactivity was evaluated in a semiquantitative manner based on both labeling intensity and the percentage of immunopositive tumor cells for all antibodies as described previously [5]. The score was calculated by multiplying the staining intensity (0 = no staining, 1 = mild staining, 2 = moderate staining, and 3 = strong staining) by the percentage of immunoreactive tumor cells (0 to 100). The immunostaining result was considered to be negative (0) when the score was < 25; weak positive (1 +) when the score was 26–100; moderate positive (2 +) when the score was 101–200; or strong positive (3 +) when the score was 201–300.

### Statistics

Results were analyzed using SPSS 19.0 software (SPSS Inc., Chicago, IL, USA). Relationships between qualitative variables were investigated using two tailed Chi-Square test or Fisher’s exact test, and quantitative variables were analyzed by t test. *P*-value of less than 0.05 was considered significant.

## Results

### Clinicopathological features

The clinicopathological features of 12 MCRN-LMPs and 33 ccRCCs with cystic component similar to MCRN-LMP are shown in Table 1. All patients with MCRN-LMP and ccRCC with cystic component similar to MCRN-LMP had no VHL syndrome (family history; retinal, cerebellar, and spinal hemangioblastomas; pheochromocytoma; pancreatic tumors and cysts; endolymphatic sac tumors) or other genetic syndromes. In our cohort of 3,265 consecutive RCCs, 12 MCRN-LMPs (partial/radical nephrectomy ratio, 9:3) were identified accounting for 0.4%, and 33 ccRCCs with cystic component similar to MCRN-LMP (partial/radical nephrectomy ratio, 18:15) were diagnosed accounting for 1.1% of 2,901 ccRCCs. The age of the patients ranged from 36 to 63 years (mean, 51 years; median, 52 years) and 34 to 71 years (mean, 52 years; median, 53 years) among 12 MCRN-LMPs and 33 ccRCCs with cystic component similar to MCRN-LMP, respectively. Eight patients were men and 4 were women (male/female ratio, 2:1) in MCRN-LMPs, and 23 patients were men and 10 were women (male/female ratio, 2.3:1) in ccRCCs with cystic component similar to MCRN-LMP. The greatest tumor diameter ranged from 1 to 8 cm (mean, 3.5 cm; median, 3 cm) and 1.5 to 10 cm (mean, 3.8 cm; median, 3.5 cm) in MCRN-LMPs and ccRCCs with cystic component similar to MCRN-LMP, respectively. Almost all MCRN-LMPs and ccRCCs with cystic component similar to MCRN-LMP were WHO/ISUP grade 1, except one MCRN-LMP and 3 ccRCCs with cystic component similar to MCRN-LMP with WHO/ISUP grade 2. The pathological stage (according to the 2018 American Joint Committee on Cancer TNM staging system) was pT1a for 10 cases (83.3%), pT2a for 2 cases (16.7%) in 12 MCRN-LMPs; and pT1a for 24 cases (72.7%), pT1b for 7 cases (21.2%), pT2a for 2 cases (6.1%) in 33 ccRCCs with cystic component similar to MCRN-LMP.

**Table 1:** Clinicopathological features of 12 MCRN-LMPs and 33 ccRCCs with cystic component similar to MCRN-LMP

Case	Age		Sex		Size (cm)		Treatment		grade (WHO/ISUP)		pT Stage		cystic percentage (%)	
	MCRN-LMP	ccRCC-M	MCRN-LMP	ccRCC-M	MCRN-LMP	ccRCC-M	MCRN-LMP	ccRCC-M	MCRN-LMP	ccRCC-M	MCRN-LMP	ccRCC-M	MCRN-LMP	ccRCC-M
1	42	34	M	M	1	3	PN	RN	2	1	pT1a	pT1a	100	90
2	47	46	F	M	3	2	PN	PN	1	1	pT1a	pT1a	100	30
3	59	56	F	M	4	10	RN	RN	1	1	pT1a	pT2a	100	90
4	55	40	F	M	8	4	PN	RN	1	1	pT2a	pT1a	100	75
5	36	64	M	F	7.5	3	RN	RN	1	1	pT2a	pT1a	100	30
6	50	36	M	F	2	2.5	PN	RN	1	1	pT1a	pT1a	100	30
7	63	60	M	F	4	4	RN	RN	1	1	pT1a	pT1a	100	50
8	43	53	M	M	3	4.5	PN	PN	1	1	pT1a	pT1b	100	90
9	56	44	M	M	2.5	4	PN	RN	1	1	pT1a	pT1a	100	80
10	52	61	M	M	3	3.5	PN	PN	1	1	pT1a	pT1a	100	70
11	52	35	M	M	2	4	PN	RN	1	1	pT1a	pT1a	100	20
12	58	59	F	F	1.5	6	PN	PN	1	1	pT1a	pT1b	100	30
13		52		M		7		RN		1		pT1b		40
14		63		M		2.5		PN		1		pT1a		25
15		50		M		2.5		PN		1		pT1a		80
16		63		M		5		PN		1		pT1b		80
17		63		M		2.5		RN		1		pT1a		90
18		41		M		3.5		PN		1		pT1a		50
19		48		F		5.5		RN		1		pT1b		90
20		51		F		4		PN		1		pT1a		90
21		57		M		1.5		PN		1		pT1a		30
22		60		M		3		PN		1		pT1a		60
23		43		F		4		PN		1		pT1a		30
24		60		M		3.5		PN		1		pT1a		90
25		67		M		2.5		RN		1		pT1a		20
26		60		M		1.5		PN		1		pT1a		50
27		36		M		4.5		RN		1		pT1b		80
28		57		F		5		RN		1		pT1b		90
29		34		F		9		PN		1		pT2a		45
30		59		M		2		RN		1		pT1a		70
31		53		M		2.5		PN		2		pT1a		50
32		71		F		2		PN		2		pT1a		30
33		46		M		2.2		PN		2		pT1a		80

*Abbreviations*: *MCRN-LMP* Multilocular cystic renal neoplasm of low malignant potential, *ccRCC* Clear cell renal cell carcinoma, *ccRCC-M* ccRCC with cystic component similar to MCRN-LMP, *PN* Partial nephrectomy, *RN* Radical nephrectomy

The comparison of clinicopathological features between 12 MCRN-LMPs and 33 ccRCCs with cystic component similar to MCRN-LMP are showed in Table 2. The results displayed that there was no significant difference in age, sex ratio, tumor size, treatment (partial/radical nephrectomy ratio), WHO/ISUP grade and pathological stage between them (*P* > 0.05).

**Table 2:** Comparison of clinicalpathological features between 12 MCRN-LMPs and 33 ccRCCs with cystic component similar to MCRN-LMP

Clinical features	MCRN-LMP	CCRCC-M	*P*
Age (years)	51	52	0.744^a^
Sex			0.846
male	8	23	
female	4	10	
Size (cm)	3.5	3.8	0.594^a^
Treatment			0.215
PN	9	18	
RN	3	15	
Grade (WHO/ISUP)			0.937
grade 1	11	30	
grade 2	1	3	
Stage (pT)			0.150
pT1a	10	24	
pT1b	0	7	
pT2a	2	2	

Chi-Square Tests for all the other analysis

*Abbreviations*: *MCRN-LMP* Multilocular cystic renal neoplasm of low malignant potential, ccRCC Clear cell renal cell carcinoma, ccRCC-M ccRCC with cystic component similar to MCRN-LMP, *PN* Partial nephrectomy, *RN* Radical nephrectomy

^a^Independent Samples T-Test

**Fig. 1:**
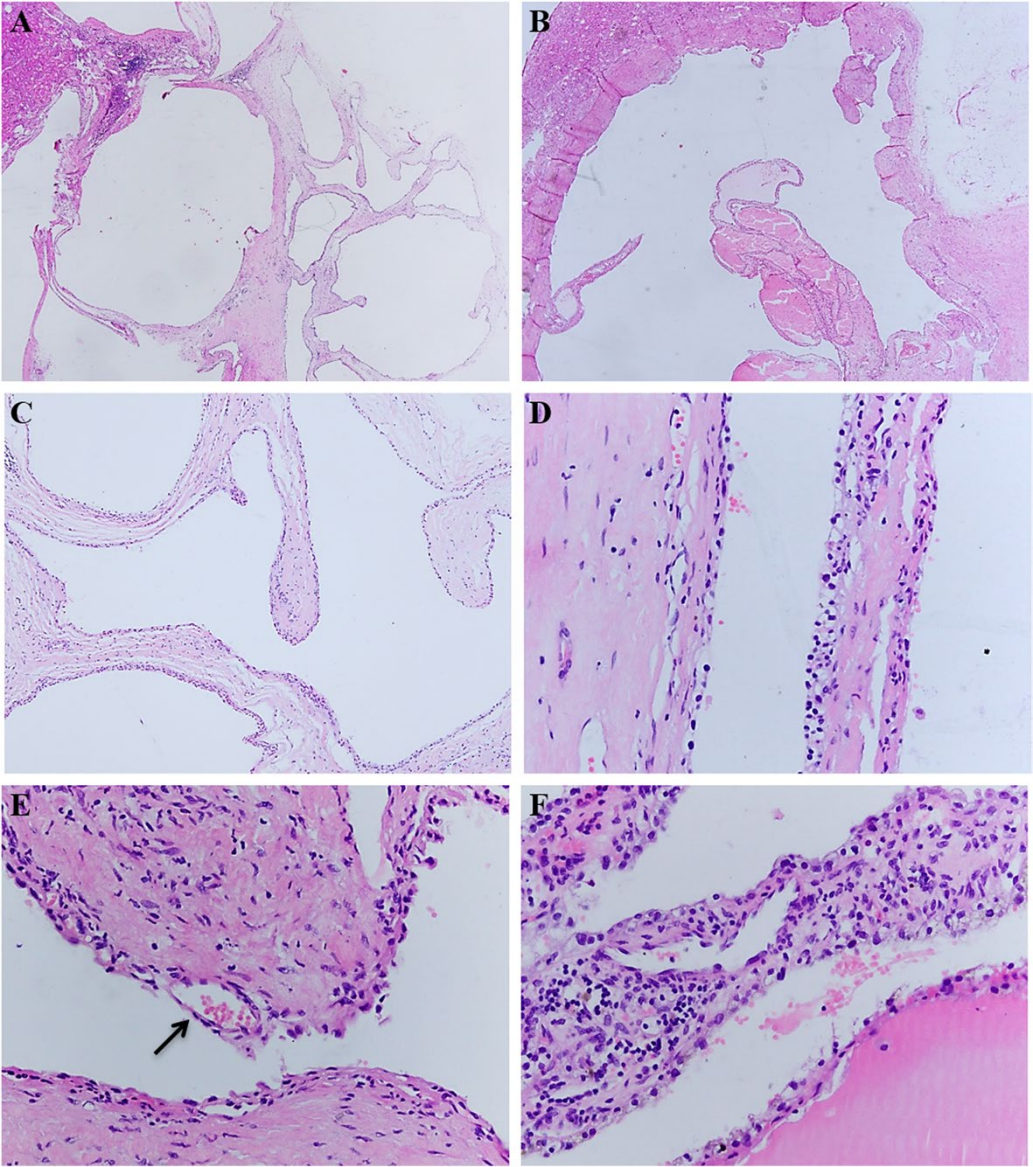
Morphologic features of multilocular cystic renal neoplasms of low malignant potential including: entirely cystic architecture **A**, **B**, thin fibrous or hyalinized septa lined by a single layer of flat to cuboidal epithelium **C** with clear cytoplasm **D** and scattered small blood vessels **E**, clear cell clusters without expansile growth **F**

**Fig. 2:**
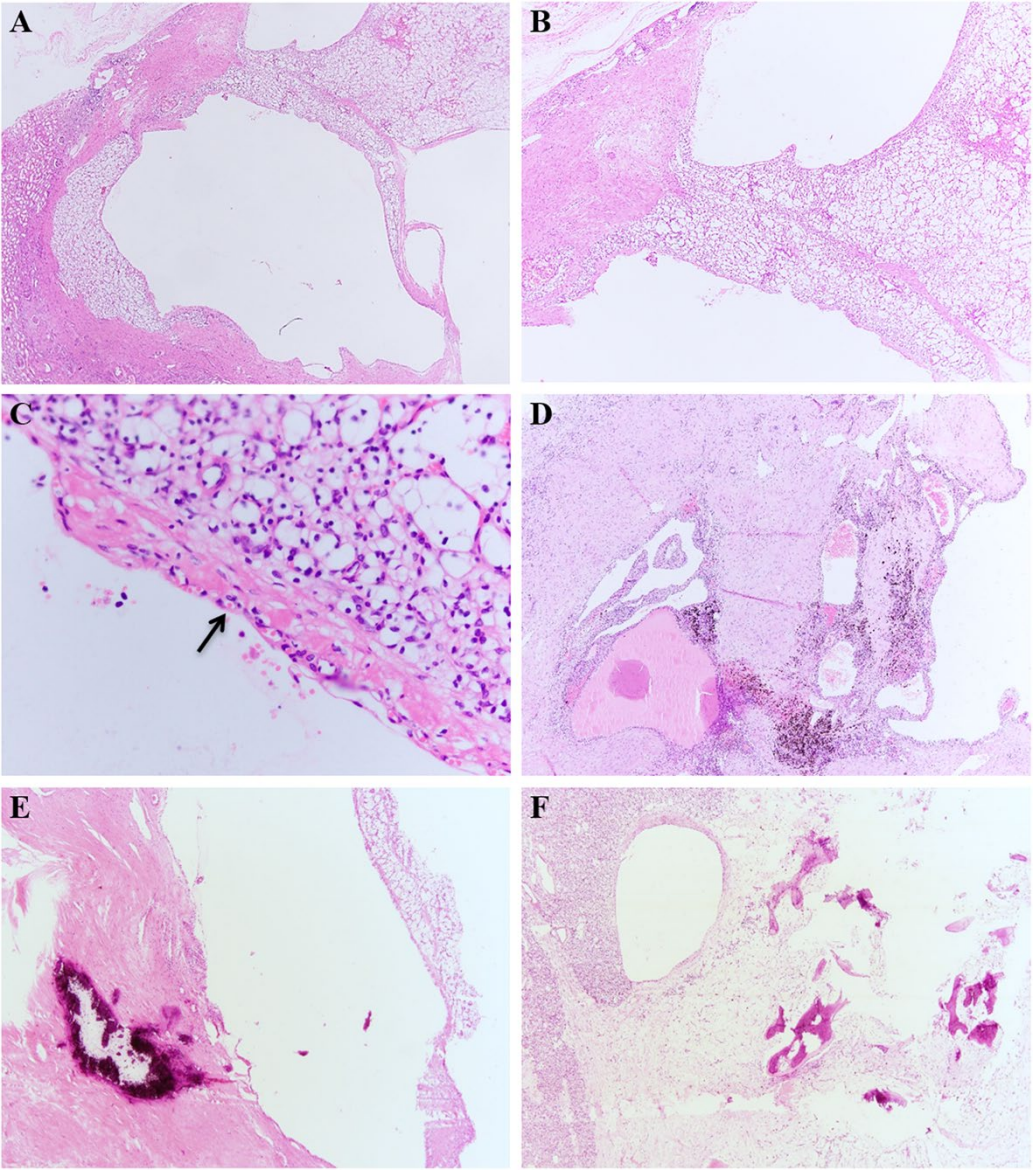
Clear cell renal cell carcinomas with cystic component similar to multilocular cystic renal neoplasm of low malignant potential coexisted with multilocular cystic renal neoplasm of low malignant potential and solid low-grade clear cell renal cell carcinoma **A**, **B**, **C**, and most of them contained foci of haemosiderin deposition **D**, and some had areas of dystrophic calcification within the hyalinized component **E**, and one had ossification **F**

The morphologic features of 12 MCRN-LMPs included an entirely cystic architecture (Fig. 1A, B), with thin fibrous or hyalinized septa lined by a single layer of flat to cuboidal epithelium (Fig. 1C), which had clear cytoplasm (Fig. 1D) and scattered small blood vessels (Fig. 1E). Occasional clusters of clear cells could be seen without expansile growth (Fig. 1F). All 33 ccRCCs with cystic component similar to MCRN-LMP coexisted with MCRN-LMP and solid low-grade ccRCCs (WHO/ISUP grade 1 or 2) (Fig. 2A, B, C). The MCRN-LMP component ranged from 20 to 90% (median, 59%) (Table 1). All tumors were free of coagulative necrosis, and most tumors contained foci of haemosiderin deposition (28/33, 84.8%) (Fig. 2D), and some had areas of dystrophic calcification within the hyalinized component (12/33, 36.4%) (Fig. 2E), and one (1/33, 3.0%) had ossification (Fig. 2F).

### IHC profiles

**Fig. 3:**
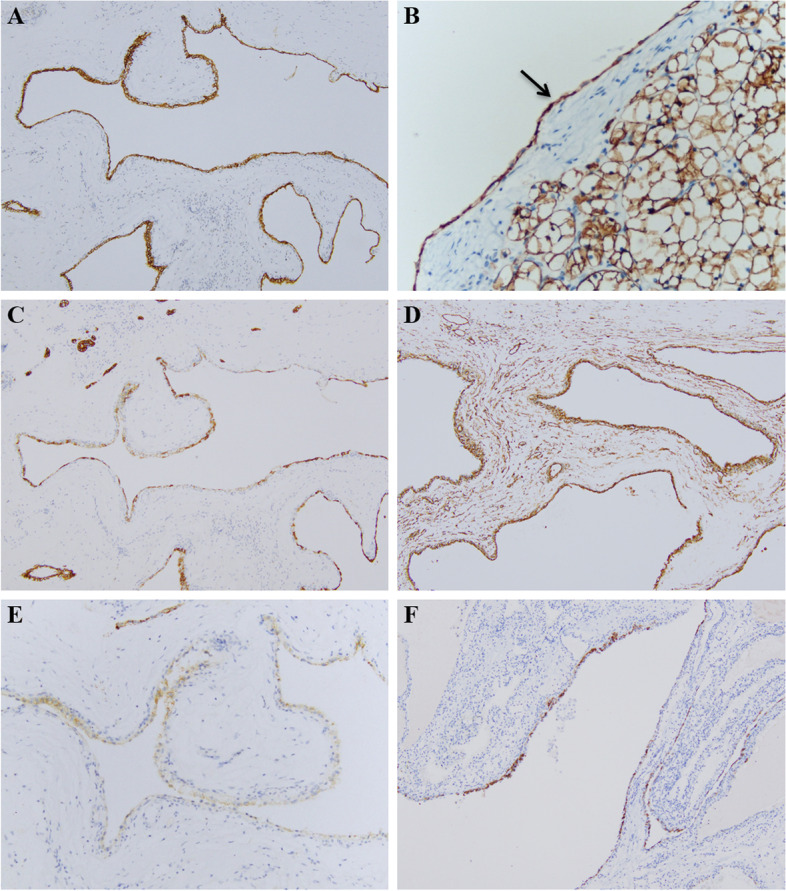
CA-IX was strong positive (3 +) staining in multilocular cystic renal neoplasms of low malignant potential **A** and clear cell renal cell carcinomas with cystic component similar to multilocular cystic renal neoplasm of low malignant potential **B**. Some immunohistochemical findings in multilocular cystic renal neoplasm of low malignant potential, including strong positive (3 +) staining for CK7 **C** and Vimentin **D**, and moderate positive (2 +) staining for P504s **E** and 34βE12 **F**

**Fig. 4:**
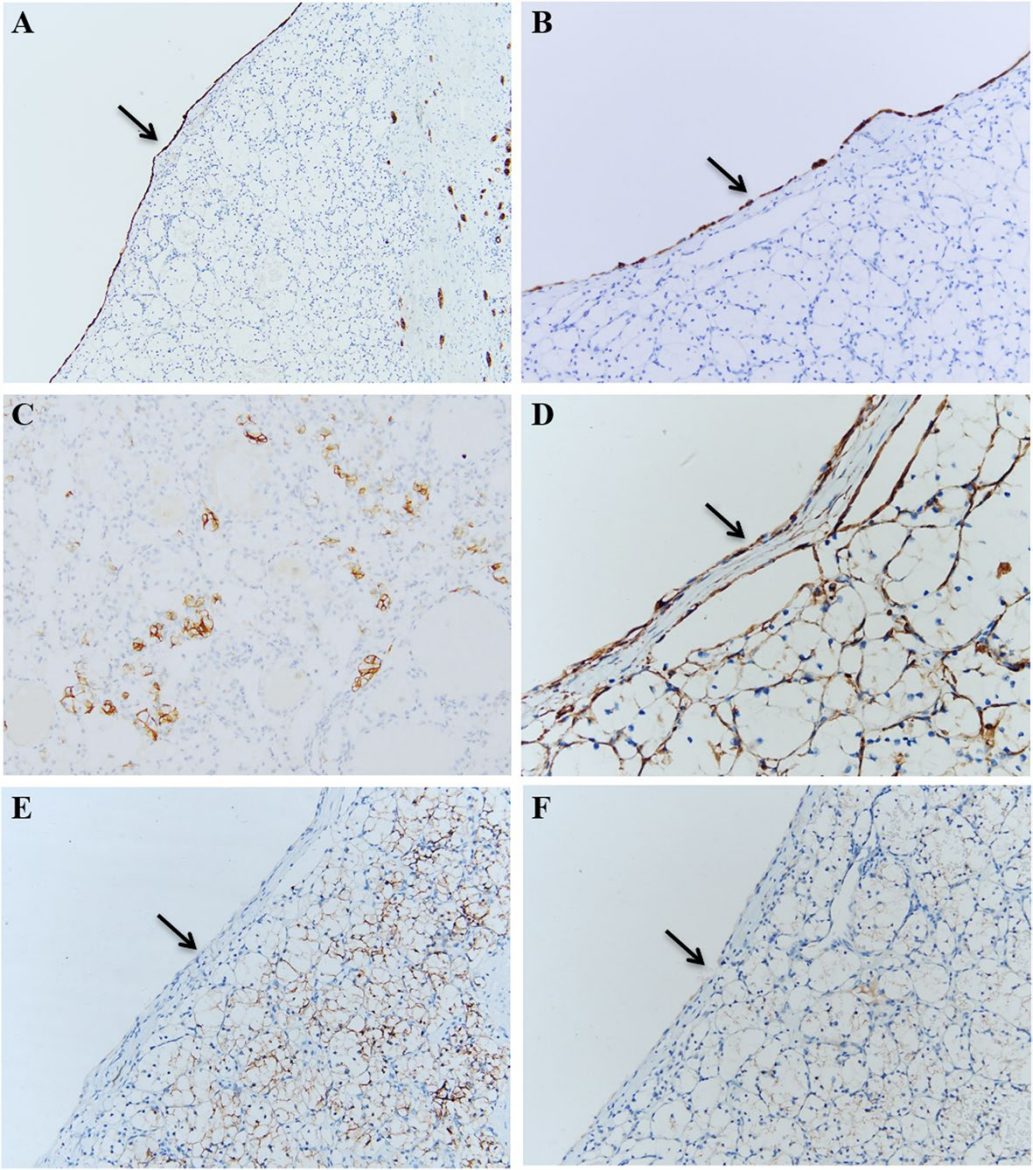
Some immunohistochemical findings in clear cell renal cell carcinomas with cystic component similar to multilocular cystic renal neoplasm of low malignant potential, including strong positive (3 +) staining in cystic parts **A**, **B** and focally positive (1 +) staining in solid parts **C** for CK7, and strong positive (3 +) staining in cystic and solid parts for Vimentin **D**, and negative staining in cystic parts **E** (arrow) and strong positive (3 +) staining in solid parts **E** for CD10, and moderate positive (2 +) staining in solid parts for P504s **F**

The IHC profiles of 12 MCRN-LMPs and 33 ccRCCs with cystic component similar to MCRN-LMP (divided into cystic part and solid part) are shown in Table 3, and the comparison of IHC findings among them are summarized in Table 4. All of 12 MCRN-LMPs and 33 ccRCCs with cystic component similar to MCRN-LMP exhibited strong positive (3 +) staining for PAX8 and CA-IX (Fig. 3A, B), but TFE3 was negative in all of them. CK7 (Fig. 3C), Vimentin (Fig. 3D), CD10, P504s (Fig. 3E) and 34βE12 (Fig. 3F) were positive in 12 (3 + , 100.0%), 12 (3 + , 100.0%), 3 (1 + , 25%), 11(6, 1 + , 50.0%; 5, 2 + , 41.7%) and 3 (2, 1 + , 16.7%; 1, 2 + , 8.3%) cases of 12 MCRN-LMPs, respectively; and they were positive in 33 (3 + , 100.0%), 31 (3 + , 93.9%), 6 (4, 1 + , 12.1%; 2, 3 + , 6.1%), 32 (16, 1 + , 48.5%; 16, 2 + , 48.5%) and 7 (1, 1 + , 3.0%; 2, 2 + , 6.1%; 4, 3 + , 12.1%) cases of 33 ccRCCs’ cystic parts (Fig. 4A, B), respectively. In addition, CK7 (Fig. 4C), Vimentin (Fig. 4D), CD10 (Fig. 4E) and P504s (Fig. 4F) were positive in 3 (2, 1 + , 6.1%; 1, 2 + , 3.0%), 32 (3 + , 97.0%), 28 (2, 1 + , 6.1%; 7, 2 + , 21.2%; 19, 3 + , 57.6%) and 33 (16, 1 + , 48.5%; 17, 2 + , 51.5%) cases of 33 ccRCCs’ solid parts, respectively, whereas 34βE12 was negative in all of them. The positive ratio of CK7 (*P2* < 0.001; *P3* < 0.001) and 34βE12 (*P2* = 0.003; *P3* = 0.006) in MCRN-LMPs and ccRCCs’ cystic parts was significantly higher than that in ccRCCs’ solid parts, but the positive ratio of CD10 in MCRN-LMPs and ccRCCs’ cystic parts was significantly lower than that in ccRCCs’ solid parts (*P2* < 0.001; *P3* < 0.001). Moreover, there was no significant difference of all IHC profiles between MCRN-LMPs and ccRCCs’ cystic parts (*P1* > 0.05).

**Table 3:** Results of immunohistochemical examination of 12 MCRN-LMPs and 33 ccRCCs with cystic component similar to MCRN-LMP

case	CK7			Vimentin			CD10			P504s			34βE12		
	MCRN-LMP	ccRCC-S	ccRCC-C	MCRN-LMP	ccRCC-S	ccRCC-C	MCRN-LMP	ccRCC-S	ccRCC-C	MCRN-LMP	ccRCC-S	ccRCC-C	MCRN-LMP	ccRCC-S	ccRCC-C
1	+ + +	+	+ + +	+ + +	-	-	-	+	-	+	+	+	-	-	-
2	+ + +	-	+ + +	+ + +	+ + +	+ + +	-	+ +	-	+	+ +	+ +	-	-	-
3	+ + +	-	+ + +	+ + +	+ + +	+ + +	-	+ + +	+ + +	-	+	-	-	-	-
4	+ + +	-	+ + +	+ + +	+ + +	+ + +	+	+ +	-	+	+ +	+ +	-	-	-
5	+ + +	-	+ + +	+ + +	+ + +	+ + +	+	+ + +	-	+	+ +	+ +	-	-	-
6	+ + +	-	+ + +	+ + +	+ + +	+ + +	-	+ +	-	+	+ +	+ +	-	-	+ + +
7	+ + +	-	+ + +	+ + +	+ + +	+ + +	-	+ + +	-	+	+ +	+ +	-	-	-
8	+ + +	-	+ + +	+ + +	+ + +	+ + +	+	+ +	-	+	+	+	-	-	-
9	+ + +	-	+ + +	+ + +	+ + +	+ + +	-	+ +	-	+	+	+	-	-	+ +
10	+ + +	-	+ + +	+ + +	+ + +	+ + +	-	+ + +	-	+	+ +	+ +	+	-	-
11	+ + +	-	+ + +	+ + +	+ + +	+ + +	-	+ + +	-	+	+ +	+ +	+	-	-
12	+ + +	-	+ + +	+ + +	+ + +	+ + +	-	+ + +	-	+ +	+	+	+ +	-	-
13		-	+ + +		+ + +	+ + +		+ + +	-		+ +	+ +		-	+
14		-	+ + +		+ + +	+ + +		+ + +	-		+ +	+ +		-	-
15		-	+ + +		+ + +	+ + +		+ +	-		+	+		-	+ +
16		-	+ + +		+ + +	+ + +		+ + +	-		+	+		-	-
17		-	+ + +		+ + +	-		+ + +	-		+	+		-	-
18		-	+ + +		+ + +	+ + +		+ + +	-		+ +	+ +		-	-
19		+ +	+ + +		+ + +	+ + +		-	-		+	+		-	-
20		-	+ + +		+ + +	+ + +		-	-		+	+		-	-
21		-	+ + +		+ + +	+ + +		+ + +	+		+ +	+ +		-	-
22		-	+ + +		+ + +	+ + +		+ + +	-		+ +	+ +		-	-
23		-	+ + +		+ + +	+ + +		+ + +	+		+ +	+ +		-	+ + +
24		-	+ + +		+ + +	+ + +		-	+		+	+		-	-
25		-	+ + +		+ + +	+ + +		+ +	-		+ +	+ +		-	+ + +
26		-	+ + +		+ + +	+ + +		+ + +	+		+ +	+ +		-	+ + +
27		-	+ + +		+ + +	+ + +		+ + +	-		+ +	+ +		-	-
28		-	+ + +		+ + +	+ + +		+ + +	-		+	+		-	-
29		-	+ + +		+ + +	+ + +		-	-		+	+		-	-
30		-	+ + +		+ + +	+ + +		-	-		+	+		-	-
31		-	+ + +		+ + +	+ + +		+ + +	-		+ +	+		-	-
32		+	+ + +		+ + +	+ + +		+	-		+	+		-	-
33		-	+ + +		+ + +	+ + +		+ + +	+ + +		+	+		-	-

*Abbreviations*: *MCRN-LMP* Multilocular cystic renal neoplasm of low malignant potential, *ccRCC* Clear cell renal cell carcinoma, *ccRCC-S* Solid part of clear cell renal cell carcinoma, *ccRCC-C* Cystic part of clear cell renal cell carcinoma

**Table 4:** Comparison of immunohistochemical findings between 12 MCRN-LMPs and 33 ccRCCs with cystic component similar to MCRN-LMP

IHC findings	0/negative (n (%))	1 + (n (%))	2 + (n (%))	3 + (n (%))	*P1*^a^	*P2*^a^	*P3*^a^
CK7					1.000	< 0.001	< 0.001
MCRN-LMP	0 (0.0)	0 (0.0)	0 (0.0)	12 (100.0)			
ccRCC-C	0 (0.0)	0 (0.0)	0 (0.0)	33 (100.0)			
ccRCC-S	30 (90.9)	2 (6.1)	1 (3.0)	0 (0.0)			
Vimentin					0.388	0.546	0.558
MCRN-LMP	0 (0.0)	0 (0.0)	0 (0.0)	12 (100.0)			
ccRCC-C	2 (6.1)	0 (0.0)	0 (0.0)	31 (93.9)			
ccRCC-S	1 (3.0)	0 (0.0)	0 (0.0)	32 (97.0)			
CD10					0.699	< 0.001	< 0.001
MCRN-LMP	9 (75.0)	3(25.0)	0 (0.0)	0 (0.0)			
ccRCC-C	27 (81.8)	4 (12.1)	0 (0.0)	2 (6.1)			
ccRCC-S	5 (15.1)	2 (6.1)	7 (21.2)	19 (57.6)			
P504s					0.591	0.420	0.719
MCRN-LMP	1 (8.3)	6 (50.0)	5 (41.7)	0 (0.0)			
ccRCC-C	1 (3.0)	16 (48.5)	16 (48.5)	0 (0.0)			
ccRCC-S	0 (0.0)	16 (48.5)	17 (51.5)	0 (0.0)			
34βE12					1.000	0.003	0.006
MCRN-LMP	9 (75.0)	2 (16.7)	1 (8.3)	0 (0.0)			
ccRCC-C	26 (78.8)	1 (3.0)	2 (6.1)	4 (12.1)			
ccRCC-S	33 (100.0)	0 (0.0)	0 (0.0)	0 (0.0)			

*Abbreviations*: *IHC* Immunohistochemical, *MCRN-LMP* Multilocular cystic renal neoplasm of low malignant potential, *ccRCC* Clear cell renal cell carcinoma, *ccRCC-S* Solid part of clear cell renal cell carcinoma, *ccRCC-C* Cystic part of clear cell renal cell carcinoma

^a^Mann-Whitney test (*P1* = MCRN-LMP: ccRCC-C; *P2* = MCRN-LMP: ccRCC-S; *P3* = ccRCC-C: ccRCC-S)

### Prognosis

Long-term follow-up was available for 12 MCRN-LMPs (range, 32–118 months; mean, 56.8 months), 33 ccRCCs with cystic component similar to MCRN-LMP (range, 16–103 months; mean, 51.2 months) from our cohort of 3,265 consecutive RCCs. All patients of 12 MCRN-LMPs and 33 ccRCCs with cystic component similar to MCRN-LMP were alive without evidence of recurrent, residual or metastatic disease at the time of most recent follow-up.

## Discussion

In this study, we reported the ratio of MCRN-LMP was 0.4% in the consecutive 3,265 RCCs, which is lower than the ratio of other studies (1–4%) [12–14], which may be because we added TFE3 immunostaining to exclude some Xp11 translocation RCCs with extensive cystic architecture similar to MCRN-LMP. For low-grade ccRCCs with a cystic component that do not meet the criteria of MCRN-LMP, the true incidence is unknown because no diagnostic terminology was clearly defined previously. Williamson et al. [7] reported 12 cases of cystic partially regressed ccRCC, comprising 3.5% of 341 ccRCCs and 2.6% of 469 RCCs. In addition, Westerman et al. [9] reported 95 cases of cystic ccRCC accounting for 2.5% of 3,865 ccRCCs. As for our cohort, the ratio of 33 ccRCCs with cystic component similar to MCRN-LMP was 1.1% of the 2,901 ccRCCs and 1.0% of the 3,265 RCCs. On the basis of these data, it is shown that both MCRN-LMP and ccRCC with cystic component similar to MCRN-LMP are all very rare, and their ratios in ccRCCs are similar, which further illustrates that their tumorigenesis may have a certain correlation. Raspollini et al. [15] conducted a genetic mutational analysis between stage pT1 ccRCCs of low ISUP/WHO nucleolar grade and MCRN-LMPs and found no significant genetic differences between them, except that a *KRAS* mutation could distinguish between the two subtypes. Furthermore, Kim et al. [16] identified six novel genetic alterations (*GIGYF2*, *FGFR3*, *SETD2*, *BCR*, *KMT2C*, and *TSC2*) that could be potential candidate genes for differentiating between MCRN-LMP and ccRCC with cystic change. As a result, we speculate that due to the overlying of other abnormal genes, some cyst‑lining cells of MCRN-LMP on the basis of *VHL* gene abnormality, further proliferate to form solid expansive nodules, and then develop into ccRCC with cystic component similar to MCRN-LMP. More studies need to be designed to prove our point, including animal model experiments.

**Fig. 5:**
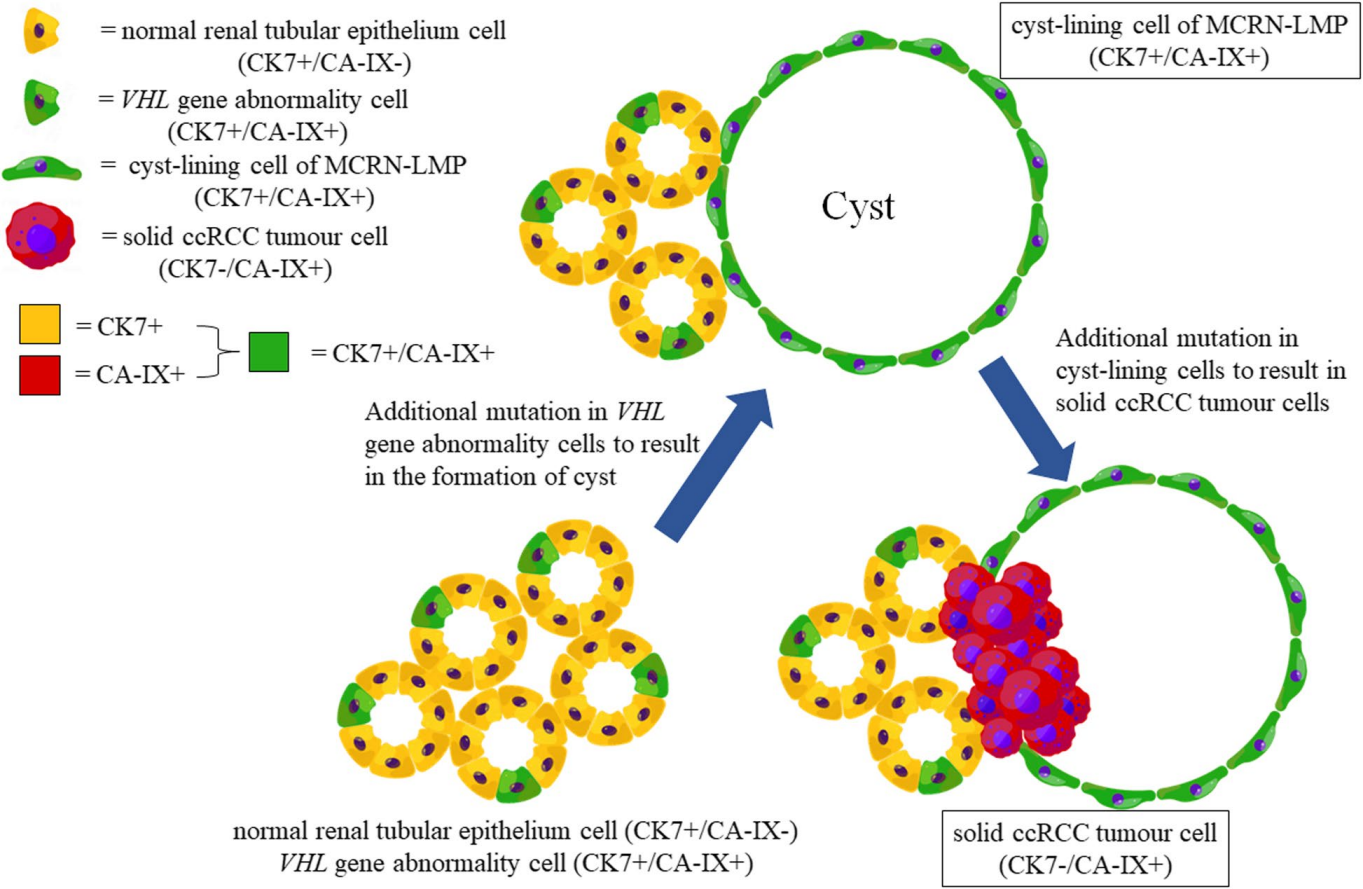
Schematic of cyst-dependent pathway from multilocular cystic renal neoplasms of low malignant potential (MCRN-LMP) to clear cell renal cell carcinoma (ccRCC) with cystic component similar to MCRN-LMP. Some normal renal tubular epithelium cells (CK7 + /CA-IX-) may progress to MCRN-LMP cyst‑lining cells (CK7 + /CA-IX +) in the case of *VHL* gene abnormality, and then may further develop into solid ccRCC tumor cells (CK7-/CA-IX +) due to the overlying of other abnormal genes

Some studies have given rise to a model of *VHL*-associated kidney disease progression in which loss of the cilia maintenance function of pVHL predisposes patients to develop cysts owing to secondary mutations that result in inactivation of GSK3β, and additional mutations in cystic cells and loss of the HIFα degradation function of pVHL are probably required for further progression from cystic precursor to ccRCC, and which suggests a cyst-dependent progression pathway of ccRCC in VHL disease [17–20]. Although our cases of MCRN-LMP and ccRCC with cystic component similar to MCRN-LMP are all sporadic patients without VHL syndrome, the relationship between them is very similar to the cyst-dependent progression pathway of ccRCC in VHL disease. Therefore, we also propose a hypothesis that the minority of sporadic ccRCCs akin to VHL disease can progress through cyst-dependent pathway from MCRN-LMP to ccRCC with cystic component similar to MCRN-LMP (Fig. 5), but the majority of sporadic ccRCCs are through cyst-independent pathway, and further research is needed to support our hypothesis.

Through clinicopathological features comparison, we noticed that there was no significant difference in age, sex ratio, tumor size, treatment (partial/radical nephrectomy ratio), WHO/ISUP grade and pathological stage between the two groups of cases, which further supports the homologous relationship between MCRN-LMP and ccRCC with cystic component similar to MCRN-LMP. In terms of morphology, the proportion of cystic component in ccRCC with cystic component similar to MCRN-LMP showed a continuous spectrum from 20 to 90%, which also provides one evidence for continuous progress from MCRN-LMP to ccRCC with cystic component similar to MCRN-LMP. Moreover, we also observed a number of morphological features associated with degeneration or regression, such as haemosiderin deposition, dystrophic calcification, hyalinized component, and ossification, which also overlap with some findings in “cystic partially regressed clear cell renal cell carcinoma” reported by Williamson et al. [7]. According to previous related studies [10, 11, 21, 22], we speculate that the low grade ccRCC with cystic component similar to MCRN-LMP through cyst-dependent pathway may be prone to degeneration or regression due to the lack of some key molecular alterations for overall tumor progression, and the specific mechanism needs to be further studied.

Our results showed that CA-IX was diffusely strong positive staining in all of 12 MCRN-LMPs and 33 cystic and solid parts of ccRCCs with cystic component similar to MCRN-LMP, which illustrates that these tumors are a subtype of ccRCC with activation of HIFα pathway due to *VHL* inactivation, as mentioned in some studies [3, 23–26]. In addition, CK7 showed diffusely strong positive staining in all MCRN-LMPs and cystic parts of ccRCCs with cystic component similar to MCRN-LMP, but often negative or focally positive in solid parts of ccRCCs with cystic component similar to MCRN-LMP. Interestingly, CK7 usually shows positive staining in some normal renal tubular epithelium [27], but is generally considered to be negative or focally positive in ccRCC [27–29], which further confirms our hypothesis of cyst-dependent pathway that some normal renal tubular epithelium cells (CK7 + /CA-IX-) may progress to MCRN-LMP cyst‑lining cells (CK7 + /CA-IX +) in the case of *VHL* gene abnormality, and then may further develop into solid ccRCC tumor cells (CK7-/CA-IX +) because of the overlying of other abnormal genes (Fig. 5).

Moreover, our results showed that CD10 was more frequently positive in solid part of ccRCCs than MCRN-LMP and cystic part of ccRCCs with cystic component similar to MCRN-LMP as some articles reported that CD10 was generally considered to be a positive marker in ccRCC [30, 31]. Furthermore, Brimo et al. [32] reported that cystic clear cell papillary RCC showed overlapping morphological features and IHC panel (positive for CA-IX, CK7, 34βE12 and negative for CD10) with MCRN-LMP, and all 9 tumors were strongly and diffusely positive for CA-IX with the pattern of cup-shaped, sparing the apical cellular portion in 8 tumors and diffuse in one, and 34βE12 expression was strong and diffuse in 8 tumors and strong but focal in one. However, our all MCRN-LMPs and ccRCCs with cystic component similar to MCRN-LMP showed diffusely strong positive for CA-IX with the pattern of box-shaped, and 34βE12 expression was strong and diffuse in 4 cystic parts of ccRCCs with cystic component similar to MCRN-LMP and mild or moderate but focal in 3 MCRN-LMPs and 3 cystic parts of ccRCCs with cystic component similar to MCRN-LMP. As a result, we think that CA-IX is the best marker for the differential diagnosis because of different expression patterns (cup-shaped and box-shaped), and once 34βE12 shows negative, it is more likely to be diagnosed as MCRN-LMP.

In this study, no patient developed recurrence or metastasis among all 12 MCRN-LMPs and 33 ccRCCs with cystic component similar to MCRN-LMP. Park et al. [33] reported that a cystic change of more than 5% of the tumor was an independent, good prognostic factor in patients with ccRCC. Han et al. [34] reported that cystic RCCs presented less often with metastatic disease and these tumors tend to be smaller, lower stage, and low grade, suggesting a more indolent biology. Webster et al. [35] reported that the estimated cancer-specific survival rate at 5 years after surgery for patients with noncystic clear cell RCC was 70.6% compared with 100% for patients with the cystic variant, and no patient with cystic ccRCC had extrarenal disease at time of nephrectomy with the exception of 1 patient who had perinephric fat invasion. Williamson et al. [7] reported that all of 16 patients of cystic partially regressed ccRCCs were alive without evidence of recurrent, residual or metastatic disease during the follow-up period from 32 to 143 months. Similarly, Tretiakova et al. [8] reported that all 69 predominantly cystic ccRCCs did not develop recurrence or metastasis with median follow-up 35.8 months (range 0–146.6), except for one contralateral kidney tumor 2 years after primary nephrectomy and one adrenal metastasis 3 years after primary diagnosis. Moreover, Westerman et al. [9] reported that all 18 MCRN-LMPs and 95 cystic ccRCCs did not develop recurrence or metastasis with median follow-up 10.3 years (interquartile range 7.4–14.9 years), except for one MCRN-LMP (contralateral recurrence) and 5 cystic ccRCCs (1 distant metastases and subsequent death from RCC at 22 years postsurgery, 1 ipsilateral and contralateral recurrence, 1 ipsilateral recurrence, and 2 contralateral recurrence), and 10- and 20-year cancer-specific survival was 100% for all cases. As a result, we think that ccRCC with cystic component similar to MCRN-LMP may have indolent or low malignant potential behavior just like MCRN-LMP, but more cases and longer follow-up time need to support this result because of the insufficiency of our number of cases and follow-up time.

Furthermore, all 12 MCRN-LMPs and 33 ccRCCs with cystic component similar to MCRN-LMP in our study were resected in a short time after their identification by imaging, which made it impossible to dynamically observe the progress of the tumors, and maybe further animal model experiments can achieve this goal. Williamson et al. [7] reported that two cystic partially regressed ccRCCs were observed with imaging prior to resection, and one remained unchanged in size over a period of 1 year, and the other enlarged over a period of 4 years and remained stable in size for the 1 year prior to resection. In addition, Jhaveri et al. [36] reported that 26 Cystic RCCs (including 13 cystic ccRCCs and 6 Multilocular cystic RCCs) were monitored with at least 6 months of pretreatment imaging, most of the tumors (73.1%) did not show a significant increase in size, and only 7 (26.9%) tumors showed growth (mean increase dimension, 10.5 mm; range, 0–24 mm). These retrospective imaging studies have shown a probable indolent course of ccRCCs with cystic component, which may also provide an ethical basis for long-term pretreatment imaging observation, and more prospective studies should be designed to detect the dynamic development of cystic RCCs, so as to further clarify the relationship between MCRN-LMPs and ccRCCs with cystic component similar to MCRN-LMP.

In summary, in this study we found that the minority (1.1%) of ccRCCs have cystic component similar to MCRN-LMP and solid low-grade component simultaneously, for which we propose the designation “ccRCC with cystic component similar to MCRN-LMP”. Further by comparing MCRN-LMP and ccRCC with cystic component similar to MCRN-LMP, we found that they have similarity and homology in clinicopathological features, immunohistochemical findings and prognosis. As a result, we speculate that MCRN-LMP and ccRCC with cystic component similar to MCRN-LMP form a low-grade spectrum with indolent or low malignant potential behavior, and ccRCC with cystic component similar to MCRN-LMP may be a rare pattern of cyst-dependent progression from MCRN-LMP. Further studies need to be designed to prove our point, including animal model experiments of molecular mechanisms and long-term pretreatment imaging observation.
